# Interrupted clinical placements among nursing students: a scoping review of causes and preventive strategies

**DOI:** 10.1186/s12912-026-05129-2

**Published:** 2026-07-28

**Authors:** Yang Li, Xiaodong Shao, Guo Wu, Yi Zhou, Li Zhou, Hongmei Yang, Yingting Wu

**Affiliations:** 1 Department of Nephrology, Guang’an People’s Hospital, Guang’an, Sichuan Province China; 2 Department of Neurology, Nanchong Central Hospital, Nanchong, Sichuan Province China

**Keywords:** Nursing students, Clinical placement, Attrition, Scoping review, Preceptor support, Peer support

## Abstract

**Aim:**

To systematically summarize the causes of interrupted clinical placements among nursing students and the strategies to prevent them, providing evidence for future research and intervention development.

**Methods:**

Following the Joanna Briggs Institute (JBI) scoping review framework, a systematic search was conducted in eight databases (CNKI, Wanfang, VIP, CBM, PubMed, Embase, Web of Science, and Cochrane Library) up to February 28, 2026. Studies examining the causes of and interventions for nursing students’ interrupted clinical placements were included.

**Results:**

A total of 23 studies were included. Causes of interruption were categorized into five themes: personal factors, clinical environment factors, socio-economic factors, demographic characteristics, and digital technology factors. The most frequently reported cause was insufficient preceptor and clinical support. Preventive strategies were grouped into five themes: psychological and emotional support, teaching and competency development, environmental and institutional optimization, preceptor and peer support, and policy and collaborative support. Preceptor and peer support was the most frequently cited strategy.

**Conclusion:**

Interruptions in clinical placements result from multifactorial interactions. Preventive strategies should be implemented collaboratively at the individual, interpersonal, environmental, and institutional levels. Preceptor and peer support may serve as a key entry point for intervention. Future research should explore longitudinal designs, digital technology interventions, and inter-institutional collaboration to establish systematic support frameworks.

**Clinical trial number:**

Not applicable.

**Supplementary Information:**

The online version contains supplementary material available at https://doi.org/10.1186/s12912-026-05129-2.

## Introduction

Global shortages of nursing staff have become a major challenge for healthcare systems, with an estimated global deficit of 5.9 million nurses by 2030 [1, 2]. Clinical placements are critical transitional stages for nursing students, bridging the gap between education and professional practice. However, clinical placements are often the most vulnerable phase of nursing education, during which students face multiple stressors, with some opting to withdraw from placements [3, 4]. Placement interruptions not only lead to a waste of educational resources but also undermine professional identity, hinder adaptation to clinical environments, and exacerbate workforce shortages [4].

Clinical placements are essential for integrating theoretical knowledge with practical skills, fostering professional identity, and building clinical confidence [5]. During this transition, students often experience a theory-practice gap, insufficient clinical competence, emotional exhaustion, and interpersonal challenges. Additionally, lack of preceptor support, high workloads, and safety concerns increase the likelihood of placement interruption [6]. Existing studies indicate that placement interruption is a multifactorial phenomenon, influenced by personal, environmental, and social factors.

Although research on nursing students’ placement interruptions has grown internationally, it remains fragmented, often focusing on single factors or isolated interventions. The role of digital technology in influencing placement continuation is emerging but remains underexplored. This scoping review, guided by the JBI framework, systematically synthesizes the literature to identify causes of placement interruption and strategies to prevent it, providing a foundation for future interventions.

## Materials and methods

### Review framework

This scoping review followed the Joanna Briggs Institute (JBI) methodology [7] and was reported according to PRISMA-ScR guidelines [8]. The protocol was registered on the Open Science Framework (OSF: https://doi.org/10.17605/OSF.IO/TFWR6).

### Review questions

1. What are the main causes of interrupted clinical placements among nursing students?
2. What strategies are effective in preventing students from discontinuing their placements?

### Inclusion and exclusion criteria

### Search strategy

A systematic search was conducted in Chinese databases (CNKI, Wanfang, VIP, CBM) and English databases (PubMed, Embase, Web of Science, Cochrane Library) up to February 28, 2026. Grey literature was searched via OpenGrey. Search terms were optimized following preliminary searches in PubMed and CNKI.The detailed search strategies for all eight databases and the grey literature search are presented in Appendix 1.

### Study selection

Retrieved records were imported into EndNote for deduplication. Two trained reviewers (Yang Li and Guo Wu) independently screened titles, abstracts, and full texts against the eligibility criteria. To assess inter-rater reliability during the screening of full-text articles, a kappa statistic was calculated based on 36 articles that underwent full-text review. The obtained kappa value was 0.76, indicating substantial agreement between the two reviewers. Disagreements occurred primarily during full-text assessment regarding the relevance of study outcomes to the review questions and were resolved through discussion with a third reviewer (Xiaodong Shao).

### Data extraction

Two reviewers independently extracted data on author, country, year, study design, causes of placement interruption, and intervention strategies. Microsoft Excel was used to manage the data extraction matrix and coding process. Differences were resolved by discussion with a third reviewer.

Following data extraction, the research team conducted a thematic categorization of the extracted content.

First, two reviewers (Yang Li and Hongmei Yang) independently read all extracted data to gain familiarity with the content.

Second, they identified and labeled meaningful units of text related to causes and strategies using an inductive open-coding approach, generating initial descriptive codes (e.g., “lack of preceptor support,” “theory–practice gap,” “peer support programs”).

Third, similar codes were grouped into preliminary categories through team discussion (e.g., “preceptor support,” “clinical environment,” “psychological factors”).

Fourth, these categories were iteratively reviewed, refined, and aggregated into overarching themes through consensus among all authors.

The themes were then mapped to the PCC framework, with nursing students as the population, placement interruption as the concept, and hospital-based clinical placements as the context.

## Results

### Literature search results

**Fig. 1:**
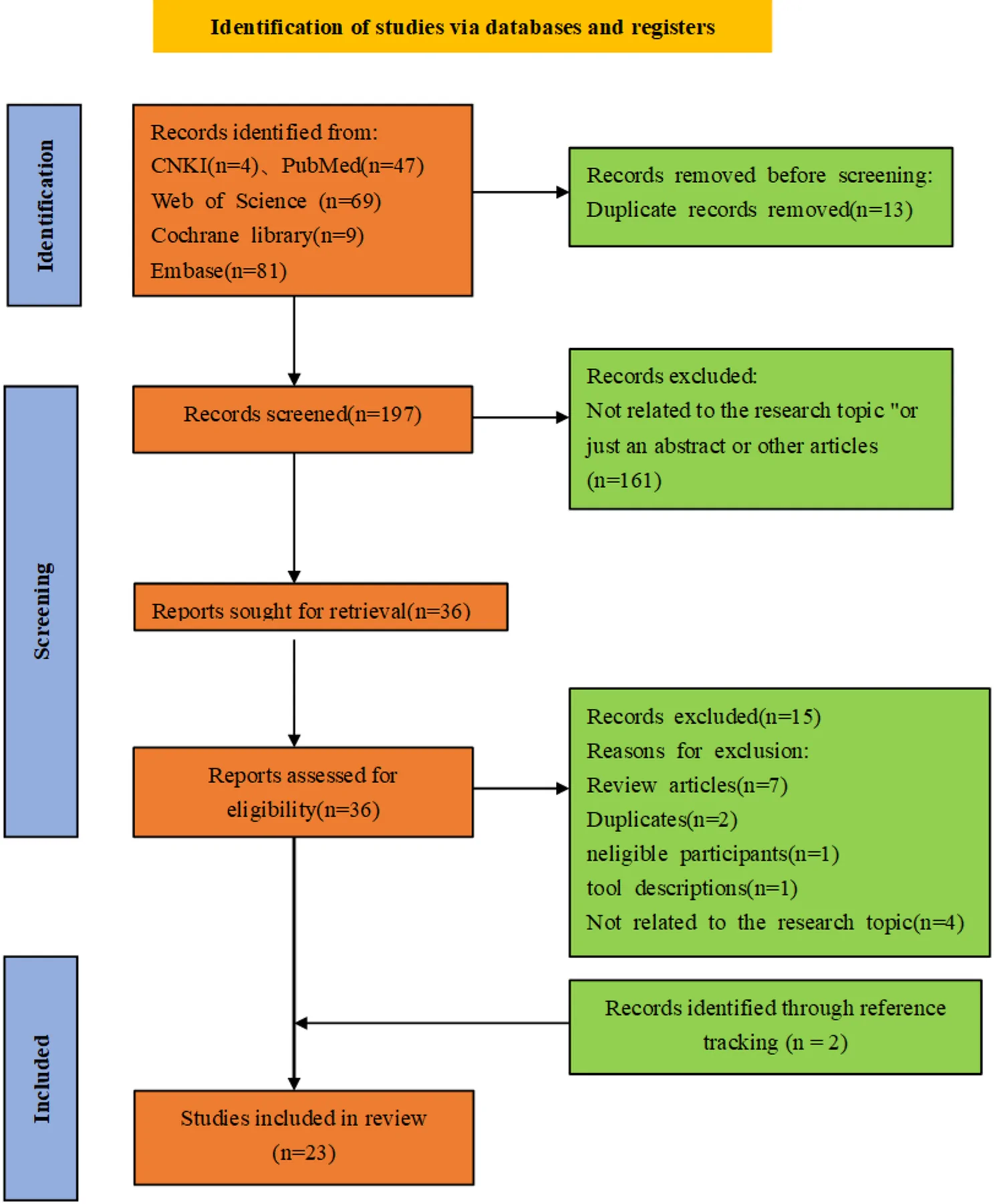
Flow chart of literature screening

An initial search yielded 210 records. After removing 13 duplicates, 197 records underwent title and abstract screening, of which 161 were excluded as irrelevant. The remaining 36 full-text articles were assessed for eligibility. Of these, 15 were excluded: seven were review articles, two were duplicate publications, one had participants not meeting the eligibility criteria, one was a methodological tool description without original data, and four were irrelevant to the research topic. Thus, 21 articles met the eligibility criteria from the database search. Two additional eligible studies were identified through manual screening of the reference lists of included articles (backward citation tracking). Ultimately, 23 studies were included in this scoping review (Fig. 1).

### Characteristics of included studies

The included studies were published between 2007 and 2025 and were conducted in China, the Netherlands, Egypt, the United Kingdom, Australia, Ireland, Denmark, Italy, and Spain. Study designs included qualitative studies, cross-sectional surveys, cohort studies, and mixed-methods studies. Sample sizes ranged from 11 to 906 participants. A summary of the study characteristics is presented in Table 1.

**Table 1:** Characteristics of included studies

First Author	Country	Year	Study Design	Sample Size
Li [9]	China	2010	Cross-sectional	146
Zhao [10]	China	2007	Cross-sectional	78
Zhong [11]	China	2015	Cross-sectional	168
Guo [12]	China	2014	Randomized Controlled	96
Ten Hoeve [13]	Netherlands	2017	Qualitative	17
Hong [14]	China	2023	Cross-sectional	275
Bakker [15]	Netherlands	2021	Prospective Cohort	363
Eltaybani [16]	Egypt	2025	Cross-sectional	906
Brook [17]	UK	2021	Qualitative	13
Bakker [18]	Netherlands	2019	Qualitative	11
Lopez [19]	Singapore	2018	Qualitative	19
Crombie [20]	UK	2013	Qualitative	50
Christiansen [21]	UK	2009	Qualitative	54
Feng [22]	China	2025	Cross-sectional	609
Molesworth [23]	UK	2017	Qualitative	17
Montgomery [24]	Australia	2025	Descriptive Cohort	47
Vasilevski [25]	Australia	2022	Qualitative	21
Thompson [26]	UK	2025	Mixed-method	540
Soerensen [27]	Denmark	2023	Qualitative	15
Sheehan [28]	Australia	2023	Mixed-method	86
Keogh [29]	Ireland	2007	Qualitative	100
Canzan [30]	Italy	2022	Qualitative	31
Arribas-Marín [31]	Spain	2021	Cross-sectional	586

### Causes of interrupted clinical placements

A thematic narrative synthesis was conducted to organize the extracted textual data, and the identified themes were mapped to the PCC elements of the JBI scoping review framework, namely participants, concept, and context. Through this process, the causal themes reflected both the frequency of reported factors and their positioning across different levels of influence. Personal factors corresponded to student-level characteristics and adaptive capacity; clinical environment factors corresponded to the immediate context in which placements occurred; socio-economic factors and demographic characteristics corresponded to external structural constraints; and digital technology factors corresponded to emerging contextual influences in contemporary clinical learning environments. Based on this synthesis, five major themes underlying nursing students’ interrupted clinical placements were identified: personal factors, clinical environment factors, socio-economic factors, demographic characteristics, and digital technology factors. Each theme and its subcategories are presented in Table 2.

#### Personal factors

Theory-practice gap, insufficient clinical competence, psychological and emotional issues, career burnout, mismatch between expectations and experience, and intentions for further study [9, 11, 13, 14, 16, 18, 30].

#### Clinical environment factors

Lack of preceptor and clinical support, difficulties with team integration, high workload, exposure to workplace violence, and unsafe team climate [9, 13, 15, 18, 19, 23, 27, 28, 30, 31].

#### Socio-economic factors

Financial pressures and early employment requirements [9, 10, 26].

#### Demographic factors

Male gender and younger age [17, 29].

#### Digital technology factors

Unmet expectations regarding digital clinical environments influencing students’ intentions to discontinue placements [22].

Among these, insufficient preceptor and clinical support was the most frequently reported cause across studies.

**Table 2:** Causes of interrupted clinical placements among nursing students

Theme	Subcategory	Specific Description	Frequency
Personal Factors	Theory–practice gap and insufficient clinical competence [9, 13, 16, 18]	①Gap between theory and practice ②Insufficient preparation for clinical practice ③Poor basic skills or inability to meet academic requirements ④ Self-perceived lack of competence	4
	Psychological / emotional issues [9, 14, 18, 30]	①High work stress / fear of errors ②Emotional exhaustion / surface acting ③Anxiety / mental collapse / insufficient psychological resources ④Personality traits amplifying negative experience	4
	Mismatch of placement experience and expectations [18, 30]	①Experience not matching expectations / career goals ②Gap between ideals and reality ③Training not aligned with expectations	2
	Career burnout and disappointment [9, 11, 30]	①Disappointment or burnout regarding nursing profession ②Low job satisfaction (fatigue, low pay, shift work) ③Awareness of unsuitability for nursing	3
	Misconception about professional knowledge [9]	①Belief that rotating a few wards suffices, learning only injections/infusions, uncertainty about future specialization	1
	Intention for further study [9, 11]	①Desire to continue education / graduate studies / degree advancement	2
Clinical Environment Factors	Lack of preceptor and clinical support [9, 13, 15, 18, 19, 23, 27, 28, 30, 31]	①Insufficient preceptor guidance; used as workforce rather than learners ②Lack of colleague or preceptor support ③Absence of clinical supervision or peer support	10
	Difficulties with team integration [13, 23, 26]	①Cannot integrate into team / marginalization ②Social isolation ③Challenges in interpersonal relationships	3
	Workload and pressure [15, 18, 19, 30]	①Overwork, dual academic-clinical pressure ②Excessive clinical workload ③Understaffed placements / high work volume ④Slow performance criticized	4
	Violence, safety, and team climate [11, 13, 15, 26, 27]	①Patient / family violence, bullying, harassment ②Poor nurse-patient relations ③ Feeling unsafe ④ Poor team atmosphere	5
Socio-Economic Factors	Financial pressure and employment needs [9, 10, 26]	①Poor family conditions; urgent need to find work ② Early employment, unpaid tuition ③ Financial stress	3
Demographic Factors	Male gender [17, 29]	①Gender discrimination, not accepted ② Differential treatment	2
	Age [17, 29]	①Younger students more likely to leave	2
Digital Technology Factors	Unmet expectations in digital clinical environment [22]	①Negative emotions arising from unmet expectations of digital tools	1

### Preventive strategies

Preventive strategies were synthesized thematically and mapped to the PCC framework according to their target levels. Psychological and emotional support and teaching and competency development mainly addressed student-level needs; preceptor and peer support addressed interpersonal and placement-level support; environmental and institutional optimization addressed the clinical context; and policy and collaborative support reflected broader institutional and system-level responses. Therefore, the frequency counts in Table 3 should be interpreted as descriptive indicators of evidence concentration rather than measures of intervention effectiveness. Based on this synthesis, preventive strategies were grouped into five major themes, with specific measures detailed in Table 3:

#### Psychological and emotional support

Counseling, resilience programs, mindfulness training, and career guidance [9, 11, 12, 14–16, 18, 19, 30].

#### Teaching and competency development

Hands-on clinical training, pre-placement preparation, simulation-based learning, and competency assessment [9, 11, 12, 16, 18, 19, 30, 31].

#### Environmental and institutional optimization

Flexible scheduling, safe clinical learning environments, violence prevention, and management policies [9, 10, 12, 15, 16, 18, 26].

#### Preceptor and peer support

Structured mentorship programs, peer learning partnerships, and supportive social networks [11, 12, 19, 20, 21, 25, 26, 28, 29, 31].

#### Policy and collaborative support

Coordination among schools, hospitals, and government, policy guidance on employment timing, and retention strategies [10, 11, 20, 26].

Preceptor and peer support was the most frequently cited intervention and appears to be the key factor in mitigating placement interruptions.

**Table 3:** Preventive strategies for nursing students’ interrupted clinical placements

Theme	Strategy	Specific Measures	Frequency
Psychological & Emotional Support	Counseling & stress management [9, 14, 15]	①Psychological counseling, talk sessions ② Mindfulness and role-play techniques ③Relaxation, cognitive refocusing, peer interaction	3
	Psychological support services [16, 18, 19]	① Accessible mental health programs ②Resilience programs (mindfulness, coping strategies)	3
	Career guidance [11, 12, 14]	① Develop career mission ②Guide realistic perception of nursing profession ③ Highlight employment advantages for male students	3
Teaching & Competency Development	Clinical skill training [9, 12, 16]	①Hands-on opportunities, one-on-one supervision ②Skills practice & competency assessment ③Tailored training for male students on emergency and technical skills	3
	Academic support [18, 31]	①Learning assistance & tutoring ②Use self-efficacy theory to guide learning objectives	2
	Pre-placement preparation [18, 19, 30]	①Introduction to ward culture ②Provide accurate career information ③Summer school for low-entry-score students	3
	Professional cognition education [11]	①Professional awareness & interest ② Life and value education	1
Environmental & Institutional Optimization	Placement scheduling [9, 17]	①Balanced rotation schedules ②Flexible shift planning (≤ 40 h/week)	2
	Environment improvement [15, 16, 18]	①Safe clinical learning environment ②Optimize staff-patient ratio, ensure rest	3
	Violence prevention [15]	① Conflict, aggression, violence training	1
	Management regulations [9, 10]	①Attendance & evaluation policies ②Select top-performing students	2
	Course optimization [26]	①Align courses with specialty needs (adult, pediatric, mental health, midwifery)	1
	Infrastructure improvement for male students [12]	① Separate uniforms, shared spaces with male staff	1
Preceptor & Peer Support	Preceptor support [11, 12, 28, 29]	①Select qualified preceptors ② Provide mentorship training & reflection opportunities	4
	Structured mentorship programs [20, 28]	①Long-term mentor-student pairing ② Assign program coordinator ③ Focus on emotional support & sense of belonging	2
	Peer support [19–21, 25, 26, 31]	①Use social media for support networks ②Peer learning partnerships ③ Peer mentoring projects	6
Policy& Collaborative Support	Multi-party collaboration [11, 20, 26]	①Communication between nursing schools & hospitals ②Regular student forums ③Joint responsibility for placement retention	3
	Policy guidance [10]	①Advise on employment timing, emphasize importance of placement	1
	Government attention [26]	①Address dropout risks, provide funding, clarify placement duration	1
	Retention strategy [26]	①Coverage across training stages, share best practices	1

## Discussion

### Overview

The findings of this review are partially consistent with those of Eick et al. [32], who demonstrated that attrition among nursing students is not attributable to a single cause but arises from the interaction of individual characteristics, placement experiences, and external contextual factors. However, unlike previous studies that mainly summarized isolated factors, the present review further identifies structural linkages among these themes. Specifically, lack of clinical support does not operate independently but may be embedded within a pathway of support deficiency, stress accumulation, and withdrawal decision-making. For instance, insufficient preceptor support may indirectly contribute to placement interruption by exacerbating psychological distress and competence anxiety.

Furthermore, Eick et al. [32] highlighted the role of placement-related push factors, such as negative placement experiences and lack of support, in influencing students’ decisions to leave. Building on this, our findings suggest that these factors are potentially modifiable and correspond closely with the most frequently reported intervention strategies, particularly preceptor and peer support. This convergence suggests that clinical support systems may represent both a major risk domain and a key leverage point for intervention.

Nevertheless, this correspondence should not be interpreted as a straightforward causal loop. Much of the existing evidence is derived from qualitative and cross-sectional studies, limiting causal inference. Therefore, the observed alignment between causes and strategies is better understood as a structural association rather than definitive causality.

### Factors contributing to interrupted clinical placements

To move beyond a simple categorization of influencing factors, this review further integrates these factors from a structural perspective. The available evidence suggests that different categories of factors do not operate independently, but interact to influence nursing students’ decisions to continue or discontinue clinical placements. For example, a lack of support within the clinical environment not only represents a direct negative experience, but may also increase psychological stress and reduce self-efficacy, thereby amplifying individual-level vulnerabilities. Similarly, socio-economic pressures may limit students’ coping resources and intensify these processes.

Accordingly, the identified factors are better understood as part of a multi-level system rather than a collection of isolated variables. Individual factors reflect students’ psychological resources and adaptive capacity, clinical environment factors represent the immediate practice context, and socio-institutional factors constitute external constraints. This structural perspective helps explain why no single factor can independently predict placement interruption and provides a more integrated basis for developing intervention strategies.

#### Clinical environment factors: lack of preceptor support and learning environment challenges

Clinical environment factors were identified as the most common causes of interrupted placements, particularly insufficient preceptor and clinical support [9, 13, 15, 18, 19, 23, 27, 28, 30]. This aligns with findings from Chen [33], indicating that the quality of the clinical learning environment significantly influences nursing students’ retention during the transition to professional practice. The clinical environment encompasses physical space, psychosocial interactions, preceptor teaching effectiveness, student engagement, and organizational culture, serving as the primary context in which students translate theory into practice and develop professional identity [34].

Current evidence suggests that nursing students frequently experience “unstructured learning opportunities,” high emotional demands, and social marginalization [35]. Although preceptors recognize the importance of developing competent nurses, time constraints, staff shortages, and limited resources often hinder the provision of adequate guidance and feedback [36]. Some students reported being treated primarily as a labor force rather than learners, being assigned tasks beyond their competence or trivial work, which further undermines the educational value of clinical placements [37]. Zhang [38] further noted that students encounter both overt pressures (e.g., excessive workload, rigid rules, lack of voice) and covert pressures (e.g., unpaid tasks, lack of respect, normalized exploitation), contributing to emotional exhaustion and weakening professional commitment.

Additionally, interpersonal challenges, integration difficulties, and exposure to violence and unsafe environments were significant barriers [11, 13, 15, 26, 27, 23]. Alshawush et al. [39] reported that 90% of nursing students experienced or witnessed workplace violence during placements. However, the fragmented communication among educational institutions, hospitals, and placement programs, combined with inadequate support systems, limited the effectiveness of violence prevention training, leading to normalized exposure and increased risk of psychological trauma, burnout, and withdrawal from placements.

#### Personal factors: psychological distress and competence anxiety

In addition to the clinical environment, personal factors were critical determinants of placement interruption [9, 13, 16, 18, 30, 11]. Nursing students often rely heavily on theoretical learning and simulation training, resulting in a theory-practice gap. Upon entering real clinical settings, students may experience complex and unpredictable patient situations, high-intensity workloads, and skill-based challenges, triggering competence anxiety, stress, emotional exhaustion, and psychological collapse [40]. Students with introverted, sensitive, or perfectionistic personality traits are particularly prone to amplifying negative experiences, which may lead to premature withdrawal [41].

Moreover, some students enter nursing programs with limited understanding of the profession, and the gap between expectations and clinical reality may reduce professional identity and commitment, ultimately leading to placement discontinuation [42]. These findings highlight the importance of providing accurate and comprehensive pre-placement information and realistic career expectations during orientation and early education.

#### Socio-economic and demographic factors

Socio-economic factors, such as family financial pressures and early employment requirements [9, 10, 26], and demographic characteristics, including male gender and younger age [17, 29], are less frequently reported in the literature but remain significant contributors to nursing students’ withdrawal from clinical placements. Financial pressure is a key real-world driver: students from economically disadvantaged families, those needing to secure employment early, or those with unpaid tuition may be compelled to leave placements prematurely to seek work [9, 10, 26]. A scoping review also highlighted that mandatory unpaid clinical placements impose additional burdens related to transportation, accommodation, and daily living expenses. The inability to earn income during placements can result in “placement poverty,” which may trigger anxiety, depression, burnout, and fatigue, disrupt students’ study–life balance, and ultimately lead to withdrawal [43].

In terms of demographic characteristics, male nursing students are highly susceptible to various forms of gender discrimination during clinical placements. Due to deeply ingrained societal stereotypes that predominantly define nursing as a female profession, male students are often marginalized within female-dominated clinical teams, facing unequal access to learning opportunities and professional recognition. Female patients and their families frequently resist intimate care provided by male interns, a phenomenon particularly pronounced in obstetric and gynecological settings. Meanwhile, clinical departments tend to assign physically demanding tasks—such as patient transfers—disproportionately to male students based on traditional gender role expectations, thereby limiting their opportunities for specialized clinical skill development. These cumulative inequities persistently undermine their sense of belonging during placements and increase their risk of premature withdrawal [29, 44]. Younger interns, due to lower psychological maturity and limited coping capacity, are even more likely to discontinue their placements [17, 29].

#### Digital technology factors

This review incorporated digital technology-related factors into the framework of factors influencing nursing students’ placement interruption, thereby extending the existing literature. However, the current evidence on this topic remains limited, with only a small number of studies examining the influence of digital clinical environments on students’ placement experiences and continuation intentions. Therefore, digital technology factors should be regarded as an emerging research area rather than a well-established major determinant.

Feng et al. [22] reported that discrepancies between nursing students’ expectations and actual experiences of digital clinical environments may influence their intention to continue placements through negative emotional responses. This suggests that digital technology is not merely a teaching or management tool, but may also represent an important contextual factor affecting students’ clinical adaptation. Particularly in the post-COVID-19 era, remote teaching, online feedback, hybrid learning, and digital workflows such as electronic health records have become increasingly common. Nursing students are therefore required not only to cope with traditional clinical pressures, but also to manage challenges related to insufficient digital competence, technology-related stress, and changes in digital communication.

Nevertheless, the available evidence remains exploratory and is insufficient to establish a clear causal relationship between digital technology factors and placement interruption. Future research should further examine the effects of digital competence, technology-related stress, digital support systems, and online feedback mechanisms on nursing students’ placement continuity, thereby strengthening the current theoretical framework of factors influencing placement interruption.

### Analysis of preventive strategies for interrupted clinical placements

#### Preceptor and peer support

This review found that preceptor and peer support is the most frequently reported strategy for preventing nursing students’ placement interruptions, with high-quality preceptor-student relationships being a key factor in student retention [29, 11, 12, 28]. Crombie et al. [20] reported that the influence of clinical preceptors on students’ intention to remain in their programs exceeds that of higher education institutions; students’ successful completion of placements largely depends on the availability of effective preceptor support during clinical experiences. When students maintain a positive relationship with their preceptors, they are more likely to persist despite academic or personal challenges. Conversely, a lack of guidance, indifferent attitudes, or barriers imposed by preceptors increases the likelihood of withdrawal.

Preceptor support is especially critical for specific groups, such as male nursing students. Keogh [29] noted that in the absence of understanding and support from preceptors, male students may encounter gender discrimination, differential treatment, or physically demanding assignments, contributing to placement withdrawal.

It is noteworthy that clinical preceptors often have an unclear understanding of their responsibilities, and the quality of guidance varies widely, limiting their ability to effectively support students [20]. Therefore, systematic and standardized preceptor training is urgently required. Training should focus on recognized core competencies, including communication skills, teaching ability, intrinsic motivation, and professional demeanor [45]. Clear preceptor selection criteria and institutional support providing dedicated time for preceptorship duties are essential [28], enabling preceptors to define their roles, improve guidance and communication, and provide professional support to ensure students successfully complete clinical placements.

In addition, peer support is another commonly cited intervention [19–21, 25, 26]. Compared with preceptor support, peer support offers inherent equality and approachability, reducing the power imbalance between students and preceptors. Students are more inclined to share genuine stress, anxiety, and difficulties with peers. Peers, having similar placement experiences, can provide contextually relevant advice that is easier to understand and accept, helping students adapt to clinical challenges and alleviate stress [46]. Palander et al. [47] found that peer support positively influences professional development (e.g., communication, empathy, critical thinking) and learning outcomes (particularly self-efficacy). The effectiveness of peer support requires structured institutional backing, including clearly defined roles, communication training, and appropriate preceptor guidance to prevent negative competition or collaboration difficulties. Overall, peer support complements preceptor guidance, is cost-effective, sustainable, and well-accepted by students. Nursing educators can implement structured peer support programs during clinical placements, with clear role definitions and training to ensure optimal outcomes.

#### Teaching and competency development

Strengthening teaching and competency development is an effective strategy to prevent placement interruptions. Interventions can be implemented in two ways: first, by enhancing clinical skills training through increased hands-on opportunities, one-on-one preceptorship, and competency assessment. Using self-efficacy theory, individualized learning goals can be developed to help students achieve their targets, thereby improving clinical competence and reducing withdrawal intentions [9, 12, 16]. Second, during pre-placement preparation, providing information about ward culture, accurate professional expectations, and realistic career information helps students form appropriate expectations [18, 19, 30]. High-fidelity simulations or simulated clinical scenarios can further enhance communication, leadership, and teamwork skills, bridging the gap between theory and practice and increasing students’ confidence in their clinical abilities, ultimately reducing the risk of placement interruption [48].

#### Environmental and institutional optimization

Environmental and institutional optimization constitutes an external intervention strategy. To address common issues such as excessive workload and exposure to workplace violence, clinical rotations can be balanced, flexible scheduling implemented, and students given a sense of control over their learning, reducing interruptions caused by overload [12]. Conflict and violence prevention training, along with established reporting and management mechanisms, can improve students’ psychological safety, reduce workload-related stress, and create a safer, more orderly clinical environment, thereby minimizing interruption risk [15, 16, 18]. However, institutional policies alone may be ineffective if not properly enforced [26]; effectiveness depends on the implementation and accountability mechanisms of healthcare institutions. Therefore, collaboration between schools and hospitals is necessary to establish safety standards, integrate violence prevention into placement quality assessments and department evaluations, and create a genuinely supportive clinical environment to prevent student withdrawal [49].

#### Psychological and emotional support

Psychological and emotional support is a critical individual-level intervention. Addressing emotional exhaustion, anxiety, and professional burnout, interventions may include counseling, reflective discussions, mindfulness training, role-play exercises, relaxation techniques, peer interaction, and resilience programs such as mindfulness-based or coping strategy courses [16, 18, 19]. These interventions aim to build psychological resources in advance, enhancing students’ resilience and emotional regulation. Additionally, fostering a sense of professional mission and guiding students to perceive the nursing profession positively [11, 12, 14] can help them maintain stable professional identity and persistence in the face of clinical stress. Notably, psychological support should be integrated with preceptor guidance and environmental interventions to more effectively reduce withdrawal risk.

#### Policy and collaborative support

Policy and collaborative support serve as macro-level strategies to ensure the effectiveness of interventions. The successful implementation of psychological support, competency development, environmental optimization, and preceptor support relies on institutional and policy frameworks. This includes enhanced communication between nursing departments and schools, regular student forums, and targeted support for students at risk of withdrawal [11, 20, 26]. Educational authorities can guide early employment decisions and emphasize the importance of completing placements [10]. Governmental attention to dropout risks, financial support policies, and clarification of placement requirements is also critical [26]. These measures collectively create a multi-level school–hospital–government support network. However, existing studies often focus on single-institution interventions, and evaluations of cross-sector collaboration remain limited, warranting further research.

## Conclusion

This scoping review systematically mapped the causes and preventive strategies for nursing students’ interrupted clinical placements. The findings indicate that placement interruption is not caused by a single factor, but results from the combined effects of personal factors, clinical environment factors, socio-economic factors, demographic characteristics, and digital technology-related factors. Among these, clinical environment factors were the most prominent, particularly insufficient preceptor and clinical support, which emerged as a key issue affecting placement continuity.

Regarding preventive strategies, preceptor and peer support was the most frequently reported intervention. Notably, both the most prominent cause of interruption and the most commonly proposed preventive strategy converged on the core dimension of clinical support. This suggests that preceptor and peer support should not be viewed as isolated measures, but rather as a central entry point for building a support system to prevent placement interruption, working together with psychological support, competency development, placement environment optimization, and school–hospital collaboration.

However, the current evidence is mainly derived from qualitative and cross-sectional studies, making it difficult to establish clear causal relationships. Digital technology-related factors also remain at an exploratory stage. Future research should employ longitudinal and interventional designs to further examine the effectiveness of preceptor support, peer support, and digital support in improving nursing students’ placement continuity.

## Limitations

This review has several limitations. First, most of the included studies were qualitative or cross-sectional in design. Although these studies provide valuable insights into nursing students’ experiences and the factors associated with placement interruption, they limit causal inference and the evaluation of intervention effectiveness. Second, although Chinese and English databases were searched and supplementary grey literature searches were conducted, potential publication bias and language limitations may still exist, as unpublished studies and studies published in other languages may have been missed. Third, the clinical placement environment has changed substantially in the post-COVID-19 era, including increased infection-control requirements, changes in placement availability and formats, greater use of online or hybrid teaching, increased psychological stress among students, and changes in clinical support mechanisms. Therefore, findings from pre- and post-COVID-19 studies may not be fully comparable, and the results of this review should be interpreted with caution. Future studies should employ longitudinal or interventional designs, compare changes in the causes of placement interruption before and after COVID-19, and evaluate the roles of digital learning, remote support, and flexible placement arrangements in the post-pandemic era.

## Supplementary Information

Below is the link to the electronic supplementary material.

Supplementary Material 1

## Data Availability

The datasets generated and/or analyzed during the current study are available from the corresponding author upon reasonable request.
