# Supplementary material for: Interrupted clinical placements among nursing students: a scoping review of causes and preventive strategies
Source: BMC Nurs. 2026 Jul 28;25:1016. doi: 10.1186/s12912-026-05129-2 (PMC13700268; doi:10.1186/s12912-026-05129-2)
Supplement: Supplementary file 1 [file 12912_2026_5129_MOESM1_ESM.docx]

**Search Strategy**

**Detailed Search Strings by Database**

**1.CNKI (China National Knowledge Infrastructure)**

| # | Search Query |
| --- | --- |
| 1 | SU = (护理实习生 + 实习护士 + 实习护生 + 临床实习护士 + 护士实习生 + 高职护理学生 + 护理本科生 + 护理学生 + 护生 + 护理专业学生) |
| 2 | SU = (实习中断 + 退出实习 + 实习流失 + 中途退出 + 终止实习 + 中止实习 + 提前结束实习 + 实习退出 + 实习脱落 + 中断实习 + 临床实习中断 + 临床实践中断 + 提前退出临床实习 + 放弃实习 + 实习弃权) |
| #1 AND #2 | 4 |

2. **WanFang Data**

| # | Search Query |
| --- | --- |
| 1 | SU =(护理实习生 OR 实习护士 OR 实习护生 OR 临床实习护士 OR 护士实习生 OR 高职护生 OR 护理本科生 OR 护理学生 OR 护生 OR 护理专业学生) |
| 2 | SU =（实习中断 OR 退出实习 OR 实习流失 OR 中途退出 OR 终止实习 OR 中止实习 OR 提前结束实习 OR 实习退出 OR 实习脱落 OR 中断实习 OR 临床实习中断 OR 临床实践中断 OR 提前退出临床实习 OR 放弃实习 OR 实习弃权) |
| #1 AND #2 | 0 |

3. **VIP (Chongqing VIP Chinese Science and Technology Periodical Database)**

| # | Search Query |
| --- | --- |
| 1 | M=(护理实习生 OR 实习护士 OR 实习护生 OR 临床实习护士 OR 护士实习生 OR 高职护生 OR 护理本科生 OR 护理学生 OR 护生 OR 护理专业学生) |
| 2 | M=（(实习中断 OR 退出实习 OR 实习流失 OR 中途退出 OR 终止实习 OR 中止实习 OR 提前结束实习 OR 实习退出 OR 实习脱落 OR 中断实习 OR 临床实习中断 OR 临床实践中断 OR 提前退出临床实习 OR 放弃实习 OR 实习弃权) |
| #1 AND #2 | 0 |

4. **CBM (Chinese Biomedical Literature Database)**

| # | Search Query |
| --- | --- |
| 1 | 学生，护理"[不加权:扩展] OR 护理实习生[常用字段:智能] OR 实习护士[常用字段:智能] OR 实习护生[常用字段:智能] OR 临床实习护士[常用字段:智能] OR 护士实习生[常用字段:智能] OR 高职护生[常用字段:智能] OR 护理本科生[常用字段:智能] OR 护理学生[常用字段:智能] OR 护生[常用字段:智能] OR 护理专业学生[常用字段:智能] |
| 2 | 临床实习[不加权:扩展] OR 实习中断[常用字段:智能] OR 退出实习[常用字段:智能] OR 实习流失[常用字段:智能] OR 中途退出[常用字段:智能] OR 终止实习[常用字段:智能] OR 中止实习[常用字段:智能] OR 提前结束实习[常用字段:智能] OR 实习退出[常用字段:智能] OR 实习脱落[常用字段:智能] OR 中断实习[常用字段:智能] OR 临床实践中断[常用字段:智能] OR 提前退出临床实习[常用字段:智能] OR 放弃实习[常用字段:智能] |
| #1 AND #2 | 0 |

**5.PubMed**

| # | Search Query |
| --- | --- |
| 1 | "Students, Nursing"[Mesh] OR "Nursing intern"[Title/Abstract] OR "Nurse intern"[Title/Abstract] OR "intern nurses"[Title/Abstract] OR "intern nursing students"[Title/Abstract] OR "clinical intern nurses"[Title/Abstract] OR "nurse interns"[Title/Abstract] OR "vocational nursing students"[Title/Abstract] OR "nursing undergraduates"[Title/Abstract] OR "nursing student"[Title/Abstract] OR "Nurses, Pupil"[Title/Abstract] OR "Nurse, Pupil"[Title/Abstract] OR "Pupil Nurse"[Title/Abstract] OR "Pupil Nurses"[Title/Abstract] |
| 2 | "Clinical placement disruption"[Title/Abstract] OR "Clinical placement attrition"[Title/Abstract] OR "premature exit from clinical training"[Title/Abstract] OR "termination of clinical placement"[Title/Abstract] OR "dropout from nursing clinical practicum"[Title/Abstract] OR "clinical practicum interruption"[Title/Abstract] OR "early withdrawal from clinical rotation"[Title/Abstract] OR "placement interruption"[Title/Abstract] OR "clinical discontinuation"[Title/Abstract] OR "attrition"[Title/Abstract] OR "intention to leave"[Title/Abstract] OR "withdrawal"[Title/Abstract] OR "discontinuation"[Title/Abstract] OR "non-completion"[Title/Abstract] |
| #1 AND #2 | 47 |

**6.Embase**

| # | Search Query |
| --- | --- |
| 1 | 'nursing student'/exp OR 'nursing intern':ti,ab,kw OR 'nurse intern':ti,ab,kw OR 'intern nurses':ti,ab,kw OR 'intern nursing students':ti,ab,kw OR 'clinical intern nurses':ti,ab,kw OR 'nurse interns':ti,ab,kw OR 'vocational nursing students':ti,ab,kw OR 'nursing undergraduates':ti,ab,kw OR 'nursing student':ti,ab,kw OR 'pupil nurse':ti,ab,kw OR 'pupil nurses':ti,ab,kw |
| 2 | 'clinical placement disruption':ti,ab,kw OR 'clinical placement attrition':ti,ab,kw OR 'premature exit':ti,ab,kw OR 'termination of clinical placement':ti,ab,kw OR 'dropout':ti,ab,kw OR 'clinical practicum interruption':ti,ab,kw OR 'early withdrawal':ti,ab,kw OR 'placement interruption':ti,ab,kw OR 'attrition':ti,ab,kw OR 'intention to leave':ti,ab,kw OR 'withdrawal':ti,ab,kw OR 'discontinuation':ti,ab,kw |
| #1 AND #2 | 81 |

7. **Web of since**

| # | Search Query |
| --- | --- |
| 1 | TOPIC: ("Students, Nursing" OR "Nursing intern" OR "Nurse intern" OR "intern nurses" OR "intern nursing students" OR "clinical intern nurses" OR "nurse interns" OR "vocational nursing students" OR "nursing undergraduates" OR "nursing student" OR "Nurses, Pupil" OR "Nurse, Pupil" OR "Pupil Nurse" OR "Pupil Nurses") |
| 2 | TOPIC: ("Clinical placement disruption" OR "Clinical placement attrition" OR "premature exit from clinical training" OR "termination of clinical placement" OR "dropout from nursing clinical practicum" OR "clinical practicum interruption" OR "early withdrawal from clinical rotation" OR "placement interruption" OR "clinical discontinuation" OR "attrition" OR "intention to leave" OR "withdrawal" OR "discontinuation" OR "non-completion") |
| #1 AND #2 | 69 |

8. **Cochrane Library**

| # | Search Query |
| --- | --- |
| 1 | [mh "Students, Nursing"] OR "nursing student*":ti,ab OR "nurse intern*":ti,ab OR "intern nurse*":ti,ab OR "pupil nurse*":ti,ab OR "vocational nursing student*":ti,ab OR "nursing undergraduate*":ti,ab OR "clinical placement":ti,ab OR "clinical practicum":ti,ab OR "clinical rotation":ti,ab OR "clinical training":ti,ab |
| 2 | "Clinical placement disruption":ti,ab OR "Clinical placement attrition":ti,ab OR "premature exit from clinical training":ti,ab OR "termination of clinical placement":ti,ab OR "dropout from nursing clinical practicum":ti,ab OR "clinical practicum interruption":ti,ab OR "early withdrawal from clinical rotation":ti,ab OR "placement interruption":ti,ab OR "clinical discontinuation":ti,ab OR "attrition":ti,ab OR "intention to leave":ti,ab OR "withdrawal":ti,ab OR "discontinuation":ti,ab OR "non-completion":ti,ab OR "drop out":ti,ab OR "drop-out":ti,ab |
| #1 AND #2 | 9 |

**9 Open Grey**

**"nursing student" AND "clinical placement interruption":0篇**
